# Greater White Matter Hyperintensity Volume Is Associated with the Number of Microhemorrhages in Preclinical Alzheimer’s Disease

**DOI:** 10.14283/jpad.2024.139

**Published:** 2024-07-24

**Authors:** Zahra Shirzadi, A. P. Schultz, M. Properzi, R. Yaari, W.-Y. W. Yau, A. M. Brickman, M. S. Rafii, M. C. Donohue, K. Ernstrom, S. Wang, C. R. Jack, S. M. Greenberg, R. Raman, P. Aisen, R. A. Sperling, J. P. Chhatwal, A4 Study teams

**Affiliations:** 1 Department of Neurology, Massachusetts General Hospital, Harvard Medical School, Boston, MA USA; 2 Eli Lilly and Company, Indianapolis, Indiana USA; 3 Taub Institute for Research on Alzheimer’s Disease and the Aging Brain, Department of Neurology, Columbia University, New York, NY USA; 4 Alzheimer’s Therapeutic Research Institute, University of Southern California, San Diego, CA USA; 5 Mayo Clinic, Radiology, Rochester, MN USA

**Keywords:** White matter hyperintensity, microhemorrhages, cerebral amyloid angiopathy, amyloid related imaging abnormalities, preclinical AD

## Abstract

**Background:**

Increased white matter hyperintensity (WMH) volume visible on MRI is a common finding in Alzheimer’s disease (AD). We hypothesized that WMH in preclinical AD is associated with the presence of advanced vessel amyloidosis manifested as microhemorrhages (MCH).

**Objectives:**

1) To assess the relationship between baseline WMH volume and baseline MCH. 2) To assess the relationship between longitudinal WMH accumulation and last MRI MCH during the double-blind phase of the A4 trial.

**Design:**

A multicenter, randomized, double-blind, placebo-controlled, Phase 3 study comparing solanezumab with placebo given as infusions once every 4 weeks over 4.5 years in subjects with preclinical AD, defined as having evidence of elevated brain amyloid before the stage of clinically evident cognitive impairment, with an optional open-label extension period.

**Setting:**

Anti-Amyloid Treatment in Asymptomatic Alzheimer’s Disease (A4) study.

**Participants:**

A sample of 1157 cognitively unimpaired older adults (mean age = 71.9 years [SD = 4.8 years], 59% women, 59% APOE ε4 carriers).

**Measurements:**

A linear regression model was used to assess the impact of baseline MCH amount (0, 1, 2+) on WMH volume. A linear mixed-effects model was used to assess the impact of last MRI MCH on longitudinal WMH. All models were corrected for age, sex, grey matter volume, cortical amyloid PET, APOE ε4 status, and treatment group.

**Results:**

Baseline WMH volume was greater in individuals with more than one MCH compared to those with no MCH (t=4.8, p<0.001). The longitudinal increase in WMH amongst individuals with one (t=2.3, p=0.025) and more than one MCH (t=6.7, p<0.001) at the last MRI was greater than those with no MCH.

**Conclusion:**

These results indicate a strong association between WMH and MCH, a common manifestation of cerebral amyloid angiopathy and ARIA-H. These results suggest that increased WMH volume may represent an early sign of vessel amyloidosis, likely prior to the emergence of MCH.

**Electronic Supplementary Material:**

Supplementary material is available in the online version of this article at 10.14283/jpad.2024.139.

## Introduction

The appearance of increased white matter hyperintensities (WMH) volume on routine magnetic resonance imaging (MRI) is very common, especially in patients with Alzheimer’s disease (AD). Prior studies have shown individuals with AD have elevated WMH volume compared to age-matched controls in both sporadic (1, 2) and autosomal dominant (3) cases. Recent studies suggest that WMH may be related to amyloid accumulation and cerebral amyloid angiopathy (CAA) (4, 5). CAA is clinically diagnosed primarily by identifying small vessel hemorrhagic lesions, most commonly, lobar cerebral microhemorrhages (MCH) (6). However, these hemorrhagic lesions are a relatively late consequence of vessel amyloidosis. The recent pathophysiologic framework of CAA (7) based on data from animal models, hereditary CAA, and sporadic CAA suggests signs of vessel damage and remodeling may be evident in the early, non-hemorrhagic phase of CAA. These non-hemorrhagic changes manifest as white matter injury, potentially including the common finding of increased WMH (7). Moreover, our recent study using data from autosomal dominant AD and older adults from Alzheimer’s Disease Neuroimaging Initiative (ADNI) revealed that WMH is elevated in individuals with evidence of CAA compared to individuals without MCH. The association of increased WMH with MCH and CAA was seen both cross-sectionally and longitudinally - independent of the effects of neurodegeneration, age, and systemic vascular risk (8).

MCH also served as a manifestation of amyloid related imaging abnormalities (ARIA) which have been associated with anti-amyloid immunotherapy of AD since early trials (9). ARIA appears in two forms, one transient and related to cerebral vasogenic edema (ARIA-E), and the other related to cerebral hemosiderin deposition (ARIA-H) (10). ARIA-H, identified most commonly by MCH, is also seen in the natural history of AD without exposure to amyloid-modifying immunotherapies likely as a result of CAA physiology (10, 11). The Anti-Amyloid Treatment in Asymptomatic AD (A4) Study is a secondary prevention trial testing solanezumab in preclinical AD (12). Given that treatment did not result in clearance of brain amyloid plaque, data from A4 allow for understanding ARIA-H in the natural history of AD in a large-scale cognitively unimpaired cohort with elevated cerebral amyloid i.e. preclinical AD. Using cross-sectional and longitudinal A4 data, we examine the following hypothesis: WMH in preclinical AD is associated with the severity of vessel amyloidosis manifested as MCH.

## Methods

### Participants

The A4 Study (NCT02008357) was conducted at 67 clinical trial sites in the United States, Canada, Japan, and Australia in cognitively unimpaired individuals (aged 65 to 85 years) with elevated amyloid as determined by florbetapir PET. Participants first underwent an initial clinic screening visit to assess cognitive and medical eligibility. Participants were considered cognitively unimpaired based on a global Clinical Dementia Rating score of 0, Mini-Mental State Exam score of 25 to 30, and Logical Memory II subscale delayed paragraph recall of the Wechsler Memory Scale-Revised score of 6 to 18. Eligible participants then underwent florbetapir PET imaging. If the PET demonstrated elevated cerebral amyloid, then a brain MRI for eligibility was conducted prior to randomization. Key exclusion criteria for participants were >4 MCH, use of AD medications, unstable anxiety or depression, or other unstable medical conditions (10). All participants provided written, informed consent prior to the performance of any study procedures, as mandated by local human subject research committees at each site.

### MRI

The following sequences acquired on 3T MRI were used in this study: 3D T1-weighted MPRAGE, axial T2*-weighted GRE, and axial T2-weighted FLAIR. We segmented WMH on FLAIR images using the HyperMapp3r algorithm (https://hypermapp3r.readthedocs.io/) (13). Subsequently, we co-registered WMH masks from FLAIR space to subject-space T1-weighted to calculate WMH volume for each session. Definite MCH were identified by experienced radiologists at Mayo Clinic (14) and were categorized by amount (0, 1, 2+). Small lesions (≤10mm) that were dissociable from small vessels were counted as definite MCH. Grey matter (GM) volume and intracranial volume were assessed using FreeSurfer 7.1.1. (https://surfer.nmr.mgh.harvard.edu/) (15). WMH was normalized to intracranial volume prior to entry into models. Normalized WMH volume was log-transformed to reduce skewness.

### PET

Florbetapir F 18 PET was acquired approximately 50 minutes after injection of 10 mCi of florbetapir F 18. Florbetapir F 18 PET images were realigned, averaged, and normalized to template space (16). We used a cortical neocortical composite SUVr referenced to the whole cerebellum (17) from pre-randomization images.

### Statistical analyses

We used a linear regression model to assess the impact of baseline MCH amount on baseline WMH volume adjusting for age, sex, GM volume, amyloid, APOE ε4 status, and treatment group. Subsequently, a linear mixed-effects regression model was used to assess the impact of last MRI MCH on longitudinal WMH adjusting for baseline age, sex, baseline grey matter volume, baseline amyloid, treatment group, and APOE ε4 status. We performed a series of sensitivity analyses on both baseline and longitudinal data. 1) we performed sensitivity analyses in which we replaced APOE ε4 status with the ε4 allele count (0, 1, and 2). 2) we added Framingham cardiovascular risk score as a covariate to the main analyses. 3) we repeated the main analyses across treatment groups in the Placebo-only group to remove any possible treatment effect. To further assess the possible effect of treatment on WMH accumulation, we used a Wilcoxon test to examine the WMH accumulation rate with respect to the treatment group.

## Results

**Figure 1:**
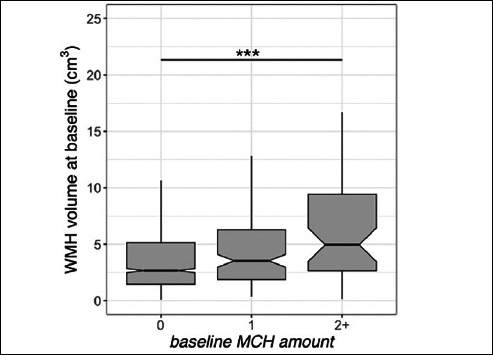
White matter hyperintensity (WMH) volume and microhemorrhages (MCH) association in A4 participants at baseline Baseline WMH volume was higher in A4 participants with more than one MCH. The middle line shows the median, the upper hinge is 75% quantile, the lower hinge is 25% quantile, lower and upper whiskers correspond to the smallest and largest observations, respectively. ***: p<0.001

**Figure 2:**
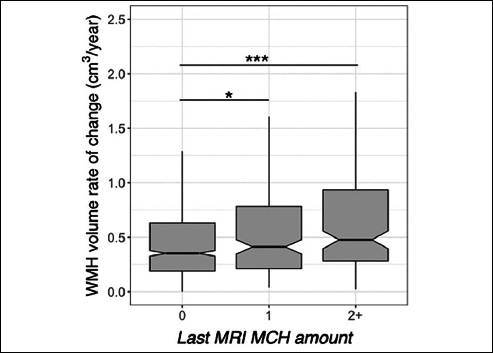
White matter hyperintensity (WMH) volume and microhemorrhages (MCH) association in A4 participants longitudinally WMH accumulation was higher in A4 participants with more than one MCH at the last MRI compared to those without. WMH rate of accumulation was higher in participants with one MCH on the last MRI compared to those without. The middle line shows the median, the upper hinge is 75% quantile, the lower hinge is 25% quantile, lower and upper whiskers correspond to the smallest and largest observations, respectively. ***: p<0.001, *:p<0.05

Data from 1157 older adults were available at the study baseline. 1130 participants had follow-up MRIs for longitudinal analysis (number of sessions=5945). Table 1 shows the participants’ demographics and imaging summary measures. We observed that baseline WMH volume was greater in individuals with more than one MCH compared to those without (t=4.8, p<0.001, Figure 1, Supplementary Table 1). Independent of the MCH effect, baseline WMH was related to older age (t=9.1, p<0.001). The longitudinal increase in WMH amongst individuals with more than one MCH on the last MRI was greater than those without (t=6.7, p<0.001, adjusted for age, sex, APOE ε4, GM volume, amyloid, and treatment group, Figure 2, Supplementary Table 3). In addition, the longitudinal increase in WMH amongst individuals with one MCH on the last MRI was greater than those without (t=2.3, p=0.025, Figure 2). We also observed a significant association between WMH accumulation and older age (t=8.9, p<0.001). Sensitivity analyses including the number of ε4 alleles did not alter these results. Sensitivity analyses including the Framingham cardiovascular risk score did not change the effects of MCH and age on WMH volume (baseline, Supplementary Table 2) or WMH accumulation (longitudinal, Supplementary Table 4). The longitudinal increase in WMH amongst placebo-only individuals with more than one MCH on the last MRI was greater than those without (t=5.8, p<0.001, adjusted for age, sex, APOE ε4, GM volume, and amyloid). In addition, there was no significant difference between treatment groups on WMH accumulation.

**Table 1:** Participants’ demographics and study information. Mean ± Standard deviation or number (percentage) are reported

	**Participants (N=1157)**
Age at baseline (years)	71.9 ± 4.8
APOE ε4 (yes)	680 (59%)
Sex (Female)	686 (59%)
Race (White /Black/Asian /other)	1088 /28 /24 /17 (94 % /2.4 % /2.1 % /1.5%)
Amyloid at baseline (PET SUVR)	1.3 ± 0.2
Definite MCH amount at baseline (0/1/2+)	944 /156 /57 (82% /13% /5%)
Definite MCH amount at the last visit (0/1/2+)	794 /186 /150 (70% /16% /14%)
Grey matter volume at baseline (cm3)	448 ± 43
Group (solanezumab /placebo)	571 /586 (49.4 % /50.6 %)
WMH volume at baseline, normalized to an intracranial volume of 1300 cm^3^	4.9 ± 6.3
Framingham cardiovascular risk score	26.8 ± 15.9
Follow up time (years)	4.43 ± 1.84

APOE ε4: Apolipoprotein E allele ε4 status; MCH: microhemorrhages; WMH: white matter hyperintensity

## Discussion

This study demonstrates an association between baseline and longitudinal WMH volume with MCH (a marker of ARIA-H and the most commonly detected manifestation of CAA). Importantly, WMH was related to MCH beyond the effects of age, sex, APOE, grey matter volume, amyloid, and systemic vascular risk. Notably, baseline WMH volume and longitudinal accumulation of WMH were related to MCH amount in this high amyloid preclinical AD cohort after accounting for the significant effect of older age. This suggests that increased WMH volume may represent an early marker for vessel amyloidosis, likely preceding the emergence of MCH.

These results are in line with prior studies from our group and others on hereditary CAA, autosomal dominant AD, and older adults with and without AD that showed elevated WMH is related to and likely precedes hemorrhagic events including MCH (8, 18, 19). Interestingly, we observed that WMH is related to having two or more MCH consistent with the diagnosis of probable CAA based on Boston criteria (6). The significance of the current study is twofold: 1) the A4 cohort represents a large population of people with elevated amyloid burden (as seen in preclinical AD), and the participants recruited into A4 resemble eligible patients to receive anti-amyloid treatments in clinical disease-modifying treatment units. These results thus shed light on understanding WMH volume and accumulation in the context of elevated amyloid burden. 2) given that treatment in A4 did not yield amyloid plaque clearing, these findings help us understand WMH progression independent of amyloid clearing in the natural history of AD and thus will provide a benchmark for AD clinical trials and treatment planning to differentiate treatment effects from white matter injury related to CAA physiology.

Of note, the global amyloid burden and APOE ε4 status did not impact WMH volume or WMH accumulation in this ‘amyloid positive’ population. This is similar to our previous findings in the autosomal dominant AD in which we did not see an association between WMH, amyloid load, and APOE ε4 (8, 20). However, we did observe an effect of amyloid load on WMH and WMH accumulation in the ADNI cohort, a study that includes both amyloid-negative and amyloid-positive individuals. We observed similar findings in the placebo-only group and combined treatment groups on the effect of MCH on WMH. Moreover, WMH accumulation was not statistically different between placebo and solanezumab groups. These findings are similar to previous reports on ARIA rates in A4 (11) and suggest that solanezumab did not have a significant effect on WMH.

This study has some limitations. We were also not able to account for the cortical/sub-cortical location of MCH in this analysis due to limited information. Given that the A4 trial did not result in amyloid reduction from baseline, this study was not able to investigate whether WMH volume or rate of accumulation are related to amyloid clearing related ARIA-H. Our results, however, show that WMH is related to ARIA-H resulting from AD natural history. Future work is needed to examine these effects in anti-amyloid drugs resulting in amyloid clearing and anti-amyloid-related ARIA. Future work will also assess WMH and WMH accumulation rate with respect to MCH in amyloid negative group, such as the LEARN cohort, to investigate WMH in a ‘non-AD’ cohort.

Taken together, these findings suggest a link between white matter injury and the number of MRI-visible microhemorrhages in individuals with elevated amyloid burden. These data support further development of white matter measures to serve as early markers of CAA. Optimized MRI-based white matter injury markers may be particularly useful to identify individuals at high risk of CAA prior to the emergence of MCH. In turn, white matter injury markers such as WMH may be useful in identifying AD patients at elevated risk of ARIA-H during the course of anti-amyloid treatment.

### Electronic Supplementary Material

Supplementary material, approximately 21.6 KB.

## Notes

*Trial Registration Information:* ClinicalTrials.gov identifier: NCT02008357.
