# Supplementary material for: Greater White Matter Hyperintensity Volume Is Associated with the Number of Microhemorrhages in Preclinical Alzheimer's Disease
Source: J Prev Alzheimers Dis. 2024 Jul 24;11(4):869–73. doi: 10.14283/jpad.2024.139 (PMC11266366; doi:10.14283/jpad.2024.139)
Supplement: Supplementary file 1 — Supplementary material, approximately 21.6 KB. [file mmc1.docx]

| **WMH volume ~** | **Estimate** | **Std. Error** | **t value** | **Pr (>\|t\|)** |
| --- | --- | --- | --- | --- |
| **(intercept)** | -27.1 | 4.2 | -6.44 | <.001 |
| **MCH (1)** | .17 | .52 | .33 | .7 |
| **MCH (2+)** | 3.97 | .83 | 4.76 | <.001 |
| **Treatment group (solanezumab)** | -.48 | .35 | -1.35 | .1 |
| **Age** | .37 | .04 | 9.1 | <.001 |
| **Sex female** | .38 | .44 | .86 | .3 |
| **Amyloid** | .82 | 1.04 | .78 | .4 |
| **Apoe e4 (yes)** | -.06 | .38 | -.15 | .9 |
| **Grey matter volume** | .01 | .005 | 1.91 | .05 |

Supplementary Table 1: results of linear regression model for baseline WMH volume.

Supplementary Table 2: results of linear regression sensitivity model for baseline WMH volume after including Framingham cardiovascular risk score.

| **WMH volume ~** | **Estimate** | **Std. Error** | **t value** | **Pr (>\|t\|)** |
| --- | --- | --- | --- | --- |
| **(intercept)** | -25.8 | 4.2 | -6.2 | <.001 |
| **MCH (1)** | .2 | .52 | .34 | .7 |
| **MCH (2+)** | 4.03 | .83 | 4.83 | <.001 |
| **Treatment group (solanezumab)** | -.41 | .35 | -1.17 | .2 |
| **age** | .3 | .04 | 7.04 | <.001 |
| **Sex female** | 1.76 | .56 | 3.13 | .002 |
| **amyloid** | .88 | 1.03 | .85 | .4 |
| **Apoe e4** | -.03 | .37 | -.09 | .9 |
| **Grey matter volume** | .01 | .005 | 2.17 | .03 |
| **Framingham cardiovascular risk score** | .06 | .02 | 3.84 | <.001 |

Supplementary Table 3: results of linear mixed effect model for longitudinal WMH volume.

| **Longitudinal WMH ~** | **Estimate** | **Std. Error** | **df** | **t value** | **Pr (>\|t\|)** |
| --- | --- | --- | --- | --- | --- |
| **(intercept)** | -30.38 | 4.85 | 1117.8 | -6.25 | <0.001 |
| **time** | .01 | .0003 | 3950.9 | 31.21 | <0.001 |
| **Last MCH (1)** | .31 | .55 | 1142.8 | .56 | .5 |
| **Last MCH (2+)** | 2.5 | .61 | 1141.8 | 4.05 | <0.001 |
| **Treatment group (solanezumab)** | -.55 | .40 | 1116.4 | -1.36 | .2 |
| **age** | .41 | .05 | 1117.9 | 8.87 | <0.001 |
| **Sex female** | .61 | .5 | 1116.1 | 1.22 | .2 |
| **amyloid** | .87 | 1.19 | 1117.4 | .73 | .4 |
| **Apoe e4** | -.19 | .43 | 1116.8 | -.45 | .6 |
| **Grey matter volume** | .01 | .006 | 1116.4 | 1.89 | .058 |
| **Time in Study** | -.002 | .003 | 1138.8 | -.88 | .3 |
| **Time * last MCH (1)** | .002 | .0007 | 3957.9 | 2.25 | .025 |
| **Time * last MCH (2+)** | .005 | .0008 | 3954.5 | 6.7 | <0.001 |

Supplementary Table 4: results of linear mixed effect sensitivity model for longitudinal WMH volume after including Framingham cardiovascular risk score.

| **Longitudinal WMH ~** | **Estimate** | **Std. Error** | **df** | **t value** | **Pr (>\|t\|)** |
| --- | --- | --- | --- | --- | --- |
| **(intercept)** | -28.9 | 4.81 | 1111.6 | -6.0 | <0.001 |
| **time** | .01 | .0003 | 3933.7 | 31.3 | <0.001 |
| **Last MCH (1)** | .37 | .55 | 1136.9 | .66 | .5 |
| **Last MCH (2+)** | 2.4 | .61 | 1135.9 | 3.87 | <0.001 |
| **Treatment group (solanezumab)** | -.50 | .40 | 1110.2 | -1.26 | .2 |
| **age** | .34 | .05 | 1111.2 | 6.86 | <0.001 |
| **Sex female** | 2.18 | .64 | 1110.6 | 3.39 | <0.001 |
| **amyloid** | 1.0 | 1.18 | 1111.1 | .84 | .4 |
| **Apoe e4** | -.17 | .43 | 1110.5 | -.41 | .7 |
| **Grey matter volume** | .01 | .006 | 1110.1 | 2.12 | .034 |
| **Time in Study** | -.002 | .003 | 1132.8 | -.71 | .5 |
| **Framingham cardiovascular risk score** | .07 | .02 | 1111.6 | 3.84 | <0.001 |
| **Time * last MCH (1)** | .002 | .0007 | 3940.8 | 2.25 | .026 |
| **Time * last MCH (2+)** | .005 | .0008 | 3937.3 | 6.37 | <0.001 |
